# Gallbladder Volvulus

**DOI:** 10.1155/1993/34905

**Published:** 1993

**Authors:** Ricardo F. Gonzalez-Fisher, Lourdes Vargas-Ramirez, Elias Rescala-Baca, Elias Dergal-Badue

**Affiliations:** 1 Department of Surgery The American-British Cowdray Hospital Mexico City Mexico; 2 Moras #306–101 Colonia del Valle Delegacion B. Juarez Mexico, D.F. 03100 Mexico

## Abstract

A case of torsion of the gallbladder is presented. This is a rare condition that occurs more frequently in elderly females, it is associated with anatomical variants related to abnormal fixation of the gallbladder to the liver bed. It is usually diagnosed at laparotomy and treatment consists of cholecystectomy. This condition should be suspected in elderly females with acute cholecystitis or acute abdominal pain of unknown origin.


*HPB Surgery*, 1993, Vol. 7, pp. 147–148
Reprints available directly from the publisher
Photocopying permitted by license only
© 1993 Harwood Academic Publishers GmbH
Printed in the United States of America

### CASE REPORT

### GALLBLADDER VOLVULUS

RICARDO F. GONZALEZ-FISHER, LOURDES VARGAS-RAMIREZ, ELIAS RESCALA-BACA and ELIAS DERGAL-BADUE

*Department of Surgery, The American-British Cowdray Hospital, Mexico City, Mexico*

*(Received 9 July 1992)*

A case of torsion of the gallbladder is presented. This is a rare condition that occurs more frequently in elderly females, it is associated with anatomical variants related to abnormal fixation of the gallbladder to the liver bed. It is usually diagnosed at laparotomy and treatment consists of cholecystectomy. This condition should be suspected in elderly females with acute cholecystitis or acute abdominal pain of unknown origin.

KEY WORDS: Gallbladder volvulus, gallbladder torsion

### INTRODUCTION

Torsion of the gallbladder is a rare entity that is usually diagnosed at laparotomy[1]. In order to avoid unnecessary complications it should be suspected in elderly patients with acute abdomen or acute cholecystitis[1,2].

### REPORT OF A CASE

A 56 year old female patient was admited with a 48 hour history of right upper quadrant abdominal pain, fever, chills and vomiting of gastric contents. She was a heavy smoker for 40 years and on medical treatment for obesity. On physical examination there was abdominal distension, right upper quadrant tenderness and a positive Murphy's sign. Laboratory studies revealed marked leucocytosis, and an abdominal ultrasonogram showed a distended gallbladder. She was rehydrated and a laparotomy was performed. The gallbladder was found to be necrotic, not attached to the liver and with a 180 degree torsion of the cystic duct and artery, no gallstones were found inside. The pedicle was detorsioned and a simple cholecystectomy was done. The patient had a favorable outcome and was discharged on the third postoperative day.

Address correspondence to: Dr Ricardo F. Gonzalez-Fisher, Moras #306–101, Colonia del Valle, Delegacion B. Juarez, 03100, Mexico, D.F.

### DISCUSSION

Since the first description of gallbladder volvulus by Wendell in 1898 there have been close to 350 recorded cases[3]. Except for isolated cases reported in children this disease is more frequent in elderly patients with a peak incidence in the 65 – 75 year old group, and a 3:1 female predominance[4].

The cause of this condition is related to anatomical variants of the gallbladder and its mesentery[1–5]. The "free floating" gallbladder or "pediculated" type in which the gallbladder is suspended from the liver by its mesentery (composed of the cystic duct and artery), the gallbladder attached to the liver with a long mesentery and the "hour glass" gallbladder in which the bottom extends freely from the liver are the most commonly described[3,4,5].

Other factors that have been implicated are ageing (by causing generalized organ ptosis), malnutrition and kyphoscoliosis[3–5]. Torsion has also been reported after a heavy meal, brisk movements, blunt trauma and post partum[1].

Approximately 50% of the patients have gallstones but it appears that this does not play a significant role in the development of the volvulus[1,3]. When rotation is less than 180 degrees, spontaneous detorsion can occur and the clinical picture will resemble that of a biliary colic. In cases of complete torsion (180 degrees or more) venous return and arterial inflow are compromised causing hemorrhagic infarction with signs and symptoms of acute cholecystitis; if the condition is unrecognized, catastrophic complications can occur, delayed treatment is associated with a 5% mortality rate[1–4].

Three cases have been diagnosed preoperatively, in two, the condition was suspected by the clinical finding of a painful mass below the liver; in the third case it was diagnosed based on ultrasonographic signs[4]; but in the majority of the cases the diagnosis is made at surgery[1–5]. Treatment consists of cholecystectomy with previous detorsion to avoid injury to the common duct[1,3,5]. Prognosis is excellent.

What is more important is to suspect this condition in elderly patients with acute cholecysitis.

### *References*

1. Smith, G.H., Perez, J.F. and Bracken, R.H. (1988) Acute torsion of the gallbladder. *Journal of the AOA*, **88**, 1523–1525
2. Ahmed, S. (1985) Torsion of the gallbladder, A case report. *SAMJ*, **68**, 614
3. Van der Veken, E., Azagra, J.S. and de Prez, C. (1986) Gallbladder volvulus: A case report. *Acta Chir. Belg.*, **86**, 267–270
4. Yeh, H-C., Weiss, M.F. and Gerson, C.D. (1989) Torsion of the gallbladder: The ultrasonographic features. *J. Clin. Ultrasound*, **17**, 123–125
5. Butsch, J.L. and Luchette, F. (1985) Torsion of the gallbladder - letter. *Arch. Surg.*, **120**, 1323

*(Accepted by S. Bengmark 2 August 1992)*

